# Clinical prognostic factors for central neurocytoma and subgroup analysis of different treatment measures: A SEER database-based retrospective analysis from 2003 to 2019

**DOI:** 10.3389/fonc.2022.1014506

**Published:** 2023-01-06

**Authors:** Zibin Zhang, Jianbo Yu, Chao Zhang, Xiaojun Pang, Yuyu Wei, Qingping Lv, Huai Chen, Xuhong Jin, Renya Zhan

**Affiliations:** ^1^ Department of Neurosurgery, Affiliated Hangzhou Chest Hospital, Zhejiang University School of Medicine, Hangzhou, China; ^2^ Department of Neurosurgery, The First Affiliated Hospital, Zhejiang University School of Medicine, Hangzhou, China

**Keywords:** central neurocytoma, SEER, prognosis, subgroup analysis, clinical application

## Abstract

**Purpose:**

The study aimed to identify clinical prognostic factors affecting overall survival (OS) in patients with central neurocytoma (CN) and to determine independent prognostic factors in the subgroups of different treatment modalities using a retrospective analysis based on the SEER database from 2003 to 2019.

**Materials and methods:**

Data regarding patients with CN, including basic clinical characteristics, treatment measures, and prognosis follow-up, were extracted from the SEER database. The prognostic variables for all patients were assessed using log-rank test as well as univariate and multivariate analyses based on the Cox proportional hazards model. The same statistical methods were used for analysis in different subgroups of gross total resection (GTR), subtotal resection (STR), no surgery, radiotherapy (RT), and no RT.

**Results:**

In total, 413 patients were enrolled in this study. Tumor size, primary site surgery, and RT were independent prognostic factors in all patients with CN. In subgroup analyses, RT was not an independent prognostic factor in patients with GTR. However, sex and race were independent prognostic factors in patients with STR. Additionally, tumor size was an independent prognostic factor in patients who did not undergo surgery. Furthermore, sex and primary site were independent prognostic factors in patients who received RT. Size and primary site surgery were independent prognostic factors in patients without RT.

**Conclusion:**

In our study, patients with small tumors or GTR or those who did not receive RT showed a better prognosis. GTR was the preferred treatment for CN. RT was not recommended for patients after GTR. Men and African American showed certain advantages after STR surgery. Tumors with a size of >4 cm were recommended for active treatment. In the RT subgroup, patients with tumors outside the ventricle or women had a poorer prognosis than those with tumors within the ventricle or men, respectively. These findings will help clinicians and patients understand the treatment and prognosis of CN visually and intuitively.

## 1 Introduction

Central neurocytoma (CN) is a rare neoplasm of the central nervous system classified as a grade II tumor by the World Health Organization (WHO) (1). It typically affects people in their 30s, which is the most common age group for the onset of cancer. CN is usually found in the ventricle system (2), and few cases have been reported in previous case reports or literature reviews. However, prognostic factors for CN remain controversial. Currently, there are limited large-scale retrospective clinical prognostic studies on CN as well as subgroup analyses of various treatment modalities.

This study aimed to identify clinical prognostic factors influencing overall survival (OS) in patients with CN and to determine independent prognostic factors in different subgroups of gross total resection (GTR), subtotal resection (STR), no surgery, radiotherapy (RT), and no RT.

## 2 Materials and methods

### 2.1 Data collection

Data regarding patients with CN, including basic clinical characteristics, social factors, tumor characteristics, treatment measures, and prognosis follow-up, were extracted from the SEER Research Plus Data (17 Registries, Nov 2021) from 2000 to 2019 using SEER*Stat software (version 8.4.0).

The inclusion criteria were as follows: (1) patients with ICD-O-3 histologic codes of 9506/0 (CN, benign), 9506/1 (CN), or 9506/3 (CN, malignant); (2) those with clear vital status and OS; and (3) those with no significant data gaps.

The exclusion criteria were as follows: (1) patients with no specific OS or OS of <1 month; (2) those with tumor locations involving other primary sites, such as the spinal cord (C72.0); or (3) those with significant data gaps or unknown mode of treatment.

**Figure 1:**
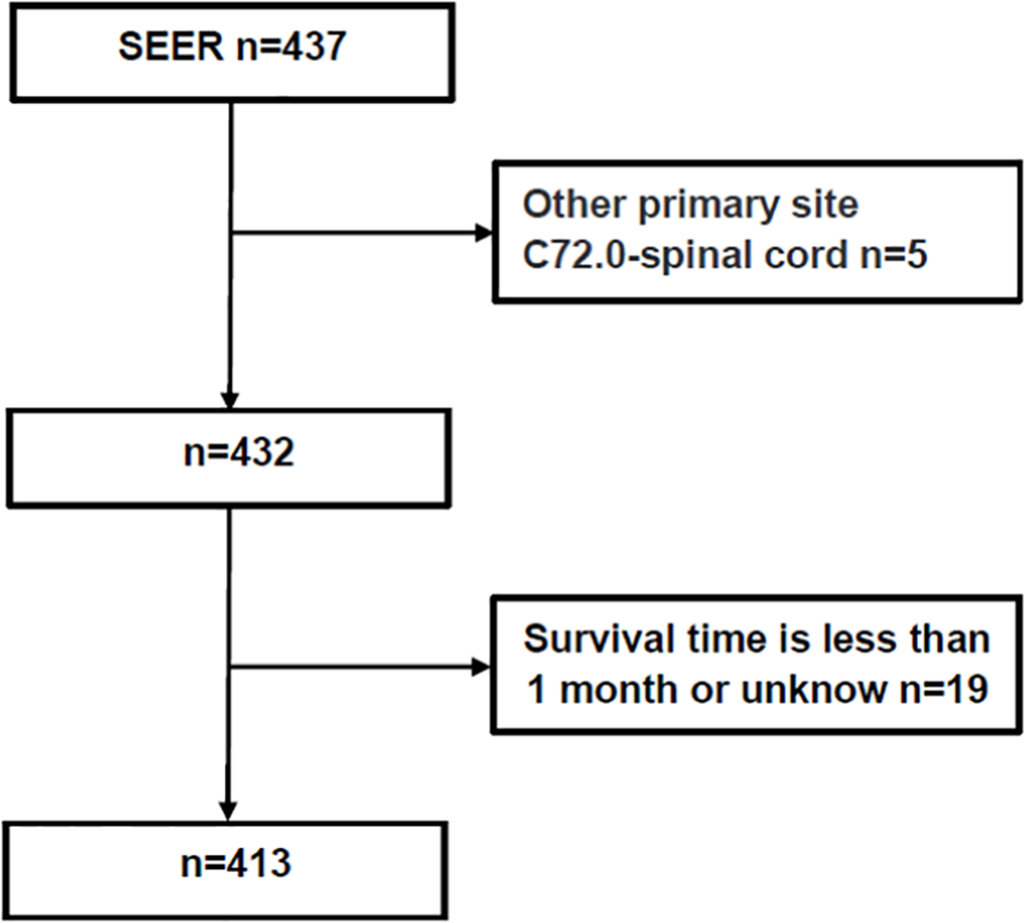
The method of obtaining data from the SEER database.

The following patient data were retrieved: age, sex, race, year of diagnosis, reporting source, primary site (location), tumor size, pathology, laterality, primary site surgery (therapy), RT, chemotherapy, vital status, and OS. GTR was defined as gross total resection of the tumor under the naked eyes or the absence of residual tumor in early postoperative imaging examination, and STR was defined as subtotal total resection of the tumor or less than 10% residual tumor under the naked eyes. RT was defined as the application of radiation to destroy or treat the primary or metastases of local tumors. In this paper, RT included simple RT, preoperative RT, intraoperative RT or postoperative RT without specific dose. The methods for obtaining data from the SEER database are described in **Figure 1**.

### 2.2 Endpoints

As the primary endpoint, OS was defined as the time from diagnosis to death or the last investigation.

### 2.3 Statistical analysis

In all patients with CN, the prognostic factors were graphically assessed using log-rank test and Kaplan–Meier curves. The independent prognostic variables were identified using univariate and multivariate analyses based on the Cox proportional hazards model. Log-rank test as well as univariate and multivariate analyses were used to identify prognostic factors in different subgroups of GTR, STR, no surgery, RT, and no RT.

Statistical analysis was performed using Statistical Package for the Social Sciences (SPSS 26.0) and R software (R 4.1.2). Factors with *P*-values of <0.10 in the univariate analysis were included in the multivariate analysis. A two-tailed *P*-value of <0.05 was considered to indicate statistical significance.

## 3 Results

### 3.1 Total data analysis

**Figure 2:**
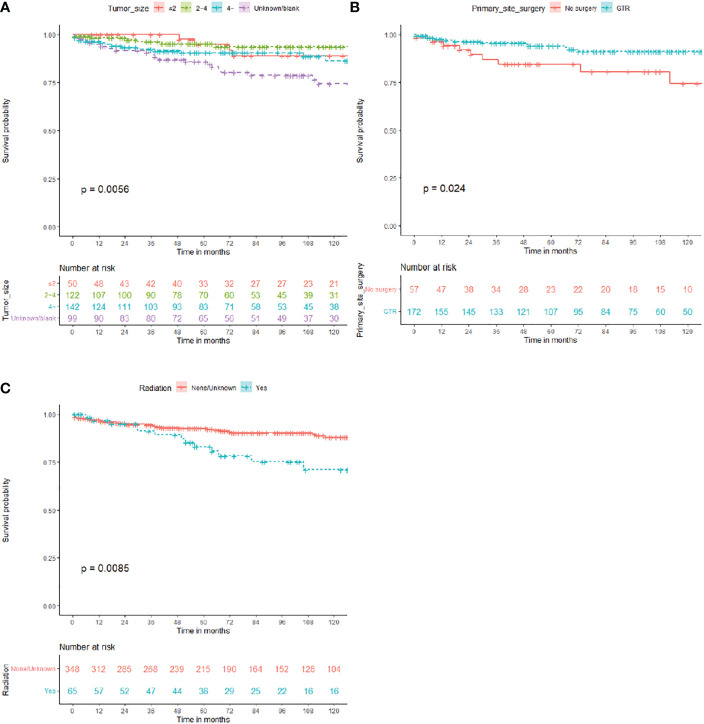
Overall survival (OS) in all patients with central neurocytoma. **(A)** OS among the different tumor size groups. **(B)** OS among the different primary site surgery (therapy) groups. **(C)** OS among the different radiation groups. OS, overall survival.

In total, 413 patients were included in this study (**Figure 1**), 203 male (49.2%) and 210 (50.8%) female. Median OS for all patients was 76 (interquartile range [IQR]: 38–128) months, 45 died (10.9%), and 368 (89.1%) survived (**Table 1**). As shown in **Figure 2**, the survival curves of tumor size (*P* = 0.0056; **Figure 2A**), primary site surgery (*P* = 0.024; **Figure 2B**), and RT (*P* = 0.0085; **Figure 2C**) were compared using log-rank test. As shown in **Table 1**, univariate analysis revealed that primary site (hazard ratio [HR]: 1.928, 95% confidence interval [CI]: 1.062–3.502, *P* = 0.031), tumor size (HR: 2.829, 95% CI: 0.971–8.245, *P* = 0.057), primary site surgery (HR: 0.384, 95% CI: 0.164–0.900, *P* = 0.028), RT (HR: 2.316, 95% CI: 1.215–4.416, *P* = 0.011), and chemotherapy (HR: 4.499, 95% CI: 1.388–14.583, *P* = 0.012) were statistically significant among the patients.

**Table 1:** Details of patients with central neurocytoma.

Characteristics		Univariate analysis			Multivariate analysis		
Total	Value N=413	HR	95%CI	*P*-value	HR	95%CI	*P*-value
Age							
0-19	61(14.8%)	Reference					
20-39	226(54.7%)	12795.377	NA	0.866			
40~59	91(22.0%)	32922.761	NA	0.853			
60~	35(8.5%)	138303.359	NA	0.833			
Sex							
Male	203(49.2%)	Reference					
Female	210(50.8%)	1.311	0.725-2.372	0.370			
Race							
White	311(75.3%)	Reference					
African American	44(10.7%)	0.565	0.175-1.832	0.342			
Others/Unknown	58(14.0%)	0.657	0.234-1.840	0.424			
Year of diagnosis							
03-11	218(52.8%)	Reference					
12-19	195(47.2%)	0.641	0.316-1.300	0.218			
Reporting Source							
Hospital inpatient/outpatient or clinic	407(98.5%)	Reference					
Other	6(1.5%)	0.049	0-60514.353	0.673			
Primary Site							
Ventricle, NOS	308(74.6%)	Reference			Reference		
Other	105(25.4%)	1.928	1.062-3.502	0.031	1.401	0.715-2.748	0.326
Tumor Size(cm)							
≤2	50(12.1%)	Reference			Reference		
2~4	122(29.5%)	0.717	0.202-2.544	0.607	1.011	0.274-3.728	0.987
4~	142(34.4%)	1.444	0.475-4.390	0.517	1.881	0.577-6.130	0.294
Unknown/blank	99(24.0%)	2.829	0.971-8.245	0.057	3.552	1.134-11.128	0.030
Pathology							
Benign	5(1.2%)	Reference					
Central neurocytoma	408(98.8%)	0.448	0.062-3.261	0.428			
Laterality							
Left-origin of primary	99(24.0%)	Reference					
Right-origin of primary	93(22.5%)	0.488	0.185-1.284	0.146			
Not a paired site	216(52.3%)	0.741	0.379-1.447	0.380			
Paired or Bilateral	5(1.2%)	0	NA	0.970			
Primary Site Surgery							
No surgery	57(13.8%)	Reference			Reference		
excisional biopsy	63(15.3%)	0.615	0.237-1.598	0.318	0.434	0.158-1.118	0.104
Surgery NOS	45(10.9%)	0.618	0.229-1.669	0.342	0.414	0.147-1.161	0.094
STR	76(18.4%)	0.651	0.250-1.693	0.379	0.471	0.169-1.310	0.149
GTR	172(41.6%)	0.384	0.164-0.900	0.028	0.298	0.122-0.728	0.008
Radiation							
None/Unknown	348(84.3%)	Reference			Reference		
Yes	65(15.7%)	2.316	1.215-4.416	0.011	2.117	1.050-4.269	0.036
Chemotherapy							
None/Unknown	407(98.5%)	Reference			Reference		
Yes	6(1.5%)	4.499	1.388-14.583	0.012	2.223	0.612-8.706	0.225
Vital Status							
Alive	368(89.1%)						
Dead	45(10.9%)						
**OS (M)**	76 (38–128)						

HR, hazard ratio; CI, confidence interval; GTR, gross total resection; STR, subtotal resection; OS, overall survival; NA, not available.

As shown in **Table 1**, multivariate analysis revealed that tumor size (HR: 3.552, 95% CI: 1.134–11.128, *P* = 0.030), primary site surgery (HR: 0.298, 95% CI: 0.122–0.728, *P* = 0.008), and RT (HR: 2.117, 95% CI: 1.050–4.269, *P* = 0.036) were independent prognostic factors in all patients with CN.

In our study, tumor size, primary site surgery, and RT were significant prognostic factors for CN. Patients with small tumors or GTR or those who did not receive RT showed a better prognosis.

### 3.2 Subgroup analysis

**Figure 3:**
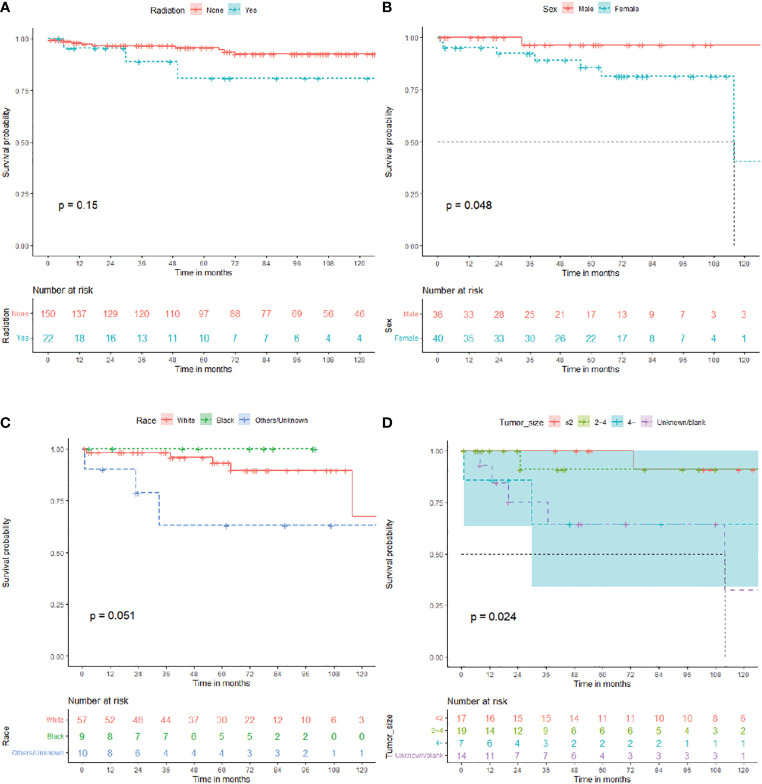
Overall survival (OS) for central neurocytoma in the gross total resection (GTR), subtotal resection (STR), and no surgery subgroups. **(A)** OS among the different radiotherapy groups in the GTR subgroup. **(B)** OS among the different sex groups in the STR subgroup. **(C)** OS among the different race groups in the STR subgroup. **(D)** OS among the different tumor size groups in the no surgery subgroup. OS, overall survival.

Overall, 172 patients with GTR were enrolled in subgroup analysis, 82 male (47.7%) and 90 female (52.3%). The median OS for patients with GTR was 81 (IQR: 40–128) months, 13 died (7.6%), and 159 (92.4%) survived (**Table 2**). As shown in **Figure 3**, the survival curves of RT (*P* = 0.15; **Figure 3A**) were compared using log-rank test. As shown in **Table 2**, univariate analysis revealed that neither RT (HR: 2.512, 95% CI: 0.689–9.165, *P* = 0.163) nor chemotherapy (HR: 0.046, 95% CI: 0–648364.957, *P* = 0.714) was statistically significant among the patients. A subgroup analysis revealed that RT did not significantly improve the prognosis of patients with GTR.

**Table 2:** The median overall survival (OS) of the gross total resection (GTR) was 81 (interquartile range (IQR): 40–128) months.

Characteristics		Univariate analysis			Multivariate analysis		
GTR	Value N=172	HR	95% CI	*P*-value	HR	95% CI	*P*-value
Age							
0-19	27(15.7%)	Reference					
20-39	108(62.8%)	17780.530	NA	0.925			
40~59	32(18.6%)	41813.135	NA	0.918			
60~	5(2.9%)	171830.274	NA	0.908			
Sex							
Male	82(47.7%)	Reference					
Female	90(52.3%)	1.052	0.353-3.135	0.927			
Race							
White	134(77.9%)	Reference					
African American	17(9.9%)	0.034	0-119.940	0.416			
Others/Unknown	21(12.2%)	0.033	0-74.877	0.387			
Year of diagnosis							
03-11	89(51.7%)	Reference					
12-19	83(48.3%)	0.812	0.238-2.775	0.740			
Reporting Source							
Hospital inpatient/outpatient or clinic	172(100.0%)						
Primary Site							
Ventricle, NOS	131(76.2%)	Reference					
Other	41(23.8%)	1.358	0.417-4.422	0.611			
Tumor Size(cm)							
≤2	15(8.7%)	Reference					
2~4	52(30.2%)	0.476	0.079-2.847	0.416			
4~	62(36.0%)	0.426	0.071-2.554	0.350			
Unknown/blank	43(25.0%)	0.839	0.163-4.327	0.834			
Pathology							
Benign	1(0.6%)	Reference					
Central neurocytoma	171(99.4%)	20.297	NA	0.868			
Laterality							
Left-origin of primary	37(21.5%)	Reference			Reference		
Right-origin of primary	44(25.6%)	0.144	0.017-1.235	0.077	0.144	0.017-1.235	0.077
Not a paired site	90(52.3%)	0.464	0.146-1.569	0.192	0.464	0.146-1.569	0.192
Paired or Bilateral	1(0.6%)	0	NA	0.989	0	NA	0.989
Radiation							
None/Unknown	150(87.2%)	Reference					
Yes	22(12.8%)	2.512	0.689-9.165	0.163			
Chemotherapy							
None/Unknown	170(98.8%)	Reference					
Yes	2(1.2%)	0.046	0-648364.957	0.714			
Vital Status							
Alive	159(92.4%)						
Dead	13(7.6%)						
**OS (M)**	81(40-128)						

HR, hazard ratio; CI, confidence interval; GTR, gross total resection; OS, overall survival; NA, not available. The Cox proportional hazards model was used for univariate and multivariate analyses in the GTR subgroup.

In total, 76 patients with STR were enrolled in subgroup analysis, 36 male (47.4%) and 40 female (52.6%). The median OS for patients with STR was 61.5 (IQR: 33.25–80) months, 8 died (10.5%), and 68 (89.5%) survived (**Table 3**). As shown in **Figure 3**, the survival curves of sex (*P* = 0.048; **Figure 3B**) and race (*P* = 0.051; **Figure 3C**) were compared using log-rank test. As presented in **Table 3**, univariate analysis revealed that sex (HR: 6.383, 95% CI: 0.780–52.215, *P* = 0.084), race (HR: 4.212, 95% CI: 0.991–17.904, *P* = 0.051), primary site (HR: 3.599, 95% CI: 0.801–16.167, *P* = 0.095), and chemotherapy (HR: 8.841, 95% CI: 0.981–79.670, *P* = 0.052) were statistically significant among the patients. As shown in **Table 3**, multivariate analysis revealed that sex (HR: 20.344, 95% CI: 1.589–260.418, *P* = 0.021) and race (HR: 13.637, 95% CI: 2.140–86.914, *P* = 0.006) were independent prognostic factors in patients with STR. In the STR subgroup, men and African American showed a better prognosis than women and other races, respectively.

**Table 3:** The median overall survival (OS) of the subtotal resection (STR) was 61.5 (interquartile range (IQR): 33.25–80) months.

Characteristics		Univariate analysis			Multivariate analysis		
STR	Value N=76	HR	95% CI	*P*-value	HR	95% CI	*P*-value
Age							
0-19	13(17.1%)	Reference					
20-39	43(56.6%)	29617.474	NA	0.945			
40~59	17(22.4%)	13186.753	NA	0.949			
60~	3(3.9%)	308576.891	NA	0.932			
Sex							
Male	36 (47.4%)	Reference			Reference		
Female	40(52.6%)	6.383	0.780-52.215	0.084	20.344	1.589-260.418	0.021
Race							
White	57(75.0%)	Reference			Reference		
African American	9(11.8%)	0	NA	0.984	0	NA	0.983
Others/Unknown	10(13.2%)	4.212	0.991-17.904	0.051	13.637	2.140-86.914	0.006
Year of diagnosis							
03-11	19(25.0%)	Reference					
12-19	57(75.0%)	0.348	0.076-1.583	0.172			
Reporting Source							
Hospital inpatient/outpatient or clinic	75(98.7%)	Reference					
Other	1(1.3%)	0.049	NA	0.890			
Primary Site							
Ventricle, NOS	61(80.3%)	Reference			Reference		
Other	15(19.7%)	3.599	0.801-16.167	0.095	5.171	0.712-37.552	0.104
Tumor Size(cm)							
≤2	5(6.6%)	Reference					
2~4	21(27.6%)	0	NA	0.962			
4~	40(52.6%)	0.615	0.068-5.520	0.664			
Unknown/blank	10(13.2%)	0.963	0.087-10.632	0.975			
Pathology							
Benign	0(0.0%)	Reference					
Central neurocytoma	76(100.0%)	NA	NA	NA			
Laterality							
Left-origin of primary	22(28.9%)	Reference					
Right-origin of primary	17(22.4%)	1.622	0.225-11.699	0.631			
Not a paired site	36(47.4%)	1.006	0.182-5.575	0.994			
Paired or Bilateral	1(1.3%)	0	NA	0.991			
Radiation							
None/Unknown	56(73.7%)	Reference					
Yes	20(26.3%)	1.571	0.373-6.612	0.538			
Chemotherapy							
None/Unknown	74(97.4%)	Reference			Reference		
Yes	2(2.6%)	8.841	0.981-79.670	0.052	2.251	0.174-29.157	0.535
Vital Status							
Alive	68(89.5%)						
Dead	8(10.5%)						
**OS (M)**	61.5(33.25-80)						

HR, hazard ratio; CI, confidence interval; STR, subtotal resection; OS, overall survival; NA, not available.

The Cox proportional hazards model was used for univariate and multivariate analyses in the STR subgroup.

Furthermore, 57 patients who did not undergo surgery were enrolled in subgroup analysis, 27 male (47.4%) and 30 female (52.6%). The median OS for patients who did not undergo surgery was 46 (IQR: 16.5–108) months, 9 died (15.8%), and 48 (84.2%) survived (**Table 4**). As shown in **Figure 3**, the survival curves of tumor sizes (*P* = 0.024; **Figure 3D**) were compared using log-rank test. As presented in **Table 4**, univariate and multivariate analyses revealed that tumor size (HR: 10.604, 95% CI: 1.216–92.460, *P* = 0.033) was an independent prognostic factor in patients without surgery. Tumors with a size of >4 cm showed a worse prognosis in patients who did not undergo surgery.

**Table 4:** The median overall survival (OS) of the no surgery subgroup was 46 (interquartile range (IQR): 16.5–108) months.

Characteristics		Univariate analysis			Multivariate analysis		
No surgery	Value N=57	HR	95% CI	*P*-value	HR	95% CI	*P*-value
Age							
0-19	4(7.0%)	Reference					
20-39	21(36.8%)	2167.939	NA	0.947			
40~59	17(29.8%)	7083.787	NA	0.938			
60~	15(26.3%)	31849.596	NA	0.928			
Sex							
Male	27 (47.4%)	Reference					
Female	30(52.6%)	0.761	0.204-2.839	0.685			
Race							
White	38(66.7%)	Reference					
African American	7(12.3%)	0.726	0.090-5.836	0.764			
Others/Unknown	12(21.1%)	0	NA	0.971			
Year of diagnosis							
03-11	26(45.6%)	Reference					
12-19	31(54.4%)	0.207	0.025-1.723	0.145			
Reporting Source							
Hospital inpatient/outpatient or clinic	55(96.5%)	Reference					
Other	2(3.5%)	0.048	NA	0.853			
Primary Site							
Ventricle, NOS	47(82.5%)	Reference					
Other	10(17.5%)	0.161	0.671-10.964	2.713			
Tumor Size(cm)							
≤2	17(29.8%)	Reference			Reference		
2~4	19(33.3%)	1.597	0.099-25.895	0.742	1.597	0.099-25.895	0.742
4~	7(12.3%)	8.076	0.722-90.344	0.090	8.076	0.722-90.344	0.090
Unknown/blank	14(24.6%)	10.604	1.216-92.460	0.033	10.604	1.216-92.460	0.033
Pathology							
Central neurocytoma	57(100.0%)	NA	NA	NA			
Laterality							
Left-origin of primary	12(21.1%)	Reference					
Right-origin of primary	16(28.1%)	0.688	0.043-11.018	0.791			
Not a paired site	28(49.1%)	2.132	0.259-17.511	0.481			
Paired or Bilateral	1(1.8%)	0	NA	0.992			
Radiation							
None/Unknown	50(87.7%)	Reference					
Yes	7(12.3%)	0749	0.094-6.004	0.786			
Chemotherapy							
None/Unknown	57(100.0%)						
Vital Status							
Alive	48(84.2%)						
Dead	9(15.8%)						
**OS (M)**	46(16.50-108)						

HR, hazard ratio; CI, confidence interval; OS, overall survival; NA, not available. The Cox proportional hazards model was used for univariate and multivariate analyses in the no surgery subgroup.

**Figure 4:**
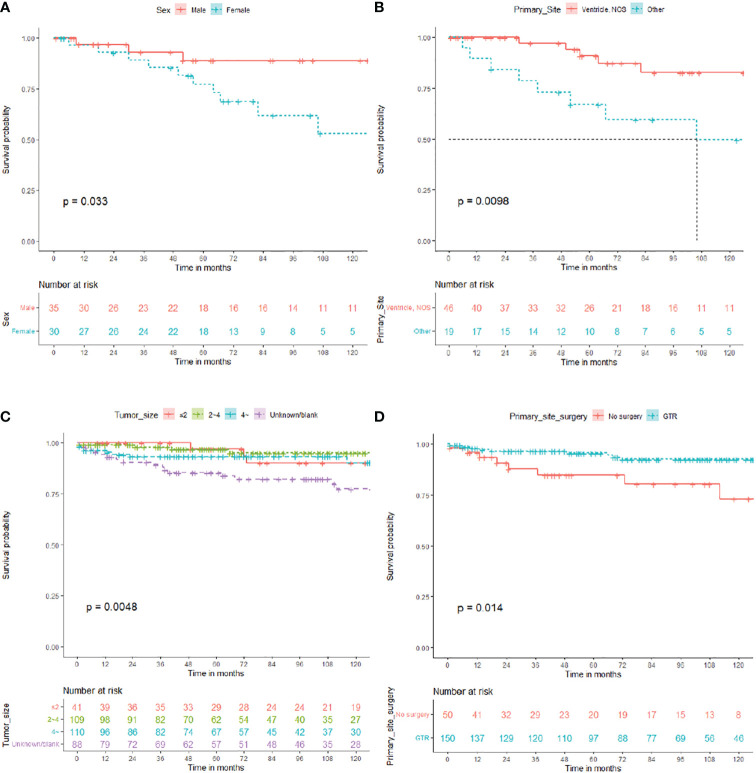
Overall survival (OS) for central neurocytoma in the radiotherapy and no radiotherapy. **(A)** OS among the different sex groups in the radiotherapy subgroup. **(B)** OS among the different primary site groups in the radiotherapy subgroup. **(C)** OS among the different tumor size groups in the no radiotherapy subgroup. **(D)** OS. among the different primary site surgery groups in the no radiotherapy subgroup. OS, overall survival.

Overall, 65 patients who received RT were enrolled in subgroup analysis, 35 male (53.8%) and 30 female (46.2%). The median OS for patients who received RT was 67 (IQR: 30–115) months, 13 died (20.0%), and 52 (80.0%) survived (**Table 5**). As shown in **Figure 4**, the survival curves of sex (*P* = 0.033; **Figure 4A**) and primary site surgery (*P* = 0.0098; **Figure 4B**) were compared using log-rank test. As depicted in **Table 5**, univariate analysis revealed that sex (HR: 3.711, 95% CI: 1.018–13.535, *P* = 0.047), primary site (HR: 3.911, 95% CI: 1.278–11.970, *P* = 0.017), and pathology (HR: 0.141, 95% CI: 0.017–1.148, *P* = 0.067) were statistically significant among these patients. As shown in **Table 5**, multivariate analysis revealed that sex (HR: 5.330, 95% CI: 1.165–24.385, *P* = 0.031) and primary site (HR: 3.472, 95% CI: 1.098–10.983, *P* = 0.034) were independent prognostic factors in patients who received RT. In the RT subgroup, patients with tumors outside the ventricle or women had a poorer prognosis than those with tumors within the ventricle or men, respectively.

**Table 5:** The median overall survival (OS) of the radiotherapy subgroup was 67 (interquartile range (IQR): 30–115) months.

Characteristics		Univariate analysis			Multivariate analysis		
Radiotherapy	Value N=65	HR	95% CI	*P*-value	HR	95% CI	*P*-value
Age							
0-19	7(10.8%)	Reference					
20-39	31(47.7%)	9815.430	NA	0.945			
40~59	23(35.4%)	27401.271	NA	0.939			
60~	4(6.2%)	126546.079	NA	0.930			
Sex							
Male	35(53.8%)	Reference			Reference		
Female	30(46.2%)	3.711	1.018-13.535	0.047	5.330	1.165-24.385	0.031
Race							
White	51(78.5%)	Reference					
African American	6(9.2%)	0.035	0-1469.791	0.537			
Others/Unknown	8(12.3%)	0.035	0-35.563	0.342			
Year of diagnosis							
03-11	31(47.7%)	Reference					
12-19	34(52.3%)	0.545	0.144-2.057	0.370			
Reporting Source							
Hospital inpatient/outpatient or clinic	64(98.5%)	Reference					
Other	1(1.5%)	0.049	NA	0.880			
Primary Site							
Ventricle, NOS	46(70.8%)	Reference			Reference		
Other	19(29.2%)	3.911	1.278-11.970	0.017	3.472	1.098-10.983	0.034
Tumor Size(cm)							
≤2	9(13.8%)	Reference					
2~4	13(20.0%)	1.403	0.127-15.526	0.783			
4~	32(49.2%)	1.746	0.210-14.530	0.606			
Unknown/blank	11(16.9%)	2.774	0.310-24.851	0.362			
Pathology							
Benign	1(1.5%)	Reference			Reference		
Central neurocytoma	64(98.5%)	0.141	0.017-1.148	0.067	0.092	0.008-1.132	0.062
Laterality							
Left-origin of primary	16(24.6%)	Reference					
Right-origin of primary	16(24.6%)	0.999	0.201-4.961	0.999			
Not a paired site	33(50.8%)	0.829	0.212-3.238	0.788			
Primary Site Surgery							
No surgery	7(10.8%)	Reference					
excisional biopsy	6(9.2%)	3.745	0.389-36.061	0.253			
Surgery NOS	10(15.4%)	1.379	0.142-13.372	0.782			
STR	20(30.8%)	1.063	0.110-10.265	0.958			
GTR	22(33.8%)	1.139	0.118-10.971	0.910			
Chemotherapy							
None/Unknown	62(95.4%)	Reference					
Yes	3(4.6%)	3.242	0.717-14.654	0.126			
Vital Status							
Alive	52(80.0%)						
Dead	13(20.0%)						
**OS (M)**	67(30-115)						

HR, hazard ratio; CI, confidence interval; GTR, gross total resection; STR, subtotal resection; OS, overall survival; NA, not available. The Cox proportional hazards model was used for univariate and multivariate analyses in the radiation subgroup.

In total, 348 patients who did not receive RT were enrolled in subgroup analysis, 168 male (48.3%) and 180 female (51.7%). The median OS for patients who did not receive RT was 79 (IQR: 38.25–132) months, 32 died (9.2%), and 316 (90.8%) survived (**Table 6**). As shown in **Figure 4**, the survival curves of tumor size (*P* = 0.0048; **Figure 4C**) and primary site surgery (*P* = 0.014; **Figure 4D**) were compared using log-rank test. As presented in **Table 6**, univariate analysis revealed that tumor size (HR: 2.922, 95% CI: 0.856–9.975, *P* = 0.087), laterality (HR: 0.999, 95% CI: 0.089–1.171, *P* = 0.085), and primary site surgery (HR: 0.329, 95% CI: 0.130–0.836, *P* = 0.019) were statistically significant among these patients. As shown in **Table 6**, multivariate analysis revealed that tumor size (HR: 3.918, 95% CI: 1.116–14.261, *P* = 0.034) and primary site surgery (HR: 0.275, 95% CI: 0.104–0.727, *P* = 0.009) were independent prognostic factors in patients without RT. In the no RT subgroup, patients with GTR showed a better prognosis.

**Table 6:** The median overall survival (OS) of the no radiotherapy subgroup was 79 (interquartile range (IQR): 38.25–132) months.

Characteristics		Univariate analysis			Multivariate analysis		
NO radiotherapy	Value N=348	HR	95% CI	*P*-value	HR	95% CI	*P*-value
Age							
0-19	54(15.5%)	Reference					
20-39	195(56.0%)	13812.672	NA	0.882			
40~59	68(19.5%)	30233.086	NA	0.872			
60~	31(8.9%)	135487.128	NA	0.854			
Sex							
Male	168(48.3%)	Reference					
Female	180(51.7%)	0.931	0.465-1.863	0.840			
Race							
White	260(74.7%)	Reference					
African American	38(10.9%)	0.793	0.239-2.630	0.705			
Others/Unknown	50(14.4%)	0.990	0.344-2.848	0.985			
Year of diagnosis							
03-11	187(53.7%)	Reference					
12-19	161(46.3%)	0.650	0.283-1.496	0.311			
Reporting Source							
Hospital inpatient/outpatient or clinic	343(98.6%)	Reference					
Other	5(1.4%)	0.049	0-478154.076	0.713			
Primary Site							
Ventricle, NOS	262(75.3%)	Reference					
Other	86(24.7%)	1.357	0.642-2.866	0.424			
Tumor Size(cm)							
≤2	41(11.8%)	Reference			Reference		
2~4	109(31.3%)	0.596	0.133-2.666	0.498	0.740	0.135-3.301	0.702
4~	110(31.6%)	1.186	0.314-4.476	0.802	1.605	0.338-6.019	0.514
Unknown/blank	88(25.3%)	2.922	0.856-9.975	0.087	3.918	1.116-14.261	0.034
Pathology							
Benign	4(1.1%)	Reference					
Central neurocytoma	344(98.9%)	20.421	NA	0.705			
Laterality							
Left-origin of primary	83(23.9%)	Reference			Reference		
Right-origin of primary	77(22.1%)	0.999	0.089-1.171	0.085	0.300	0.081-1.110	0.071
Not a paired site	183(52.6%)	0.829	0.331-1.543	0.392	0.726	0.328-1.605	0.428
Paired or Bilateral	5(1.4%)	0	0	0.981	0	0	0.982
Primary Site Surgery							
No surgery	50(14.4%)	Reference			Reference		
excisional biopsy	57(16.4%)	0.417	0.136-1.279	0.126	0.307	0.096-0.983	0.047
Surgery NOS	35(10.1%)	0.466	0.139-1.560	0.216	0.376	0.110-1.286	0.119
STR	56(16.1%)	0.560	0.182-1.719	0.311	0.568	0.169-1.905	0.360
GTR	150(43.1%)	0.329	0.130-0.836	0.019	0.275	0.104-0.727	0.009
Chemotherapy							
None/Unknown	345(99.1%)	Reference					
Yes	3(0.9%)	3.870	0.527-28.410	0.183			
Vital Status							
Alive	316(90.8%)						
Dead	32(9.2%)						
**OS (M)**	79(38.25-132)						

HR, hazard ratio; CI, confidence interval; GTR, gross total resection; STR, subtotal resection; OS, overall survival; NA, not available. The Cox proportional hazards model was used for univariate and multivariate analyses in the no radiotherapy subgroup.

## 4 Discussion

In 1982, Hassoun et al. identified two cases of tumors originating in the third ventricle and named them as CN (3). CN is a rare intracranial tumor that accounts for 0.1%–0.5% of all intracranial tumors and is classified as a grade II tumor by the World Health Organization in 2021 (1, 4, 5). CN commonly occurs in the lateral ventricle but is also found in the posterior fossa or other locations (6). Its pathogenesis is associated with various chromosomal aberrations (7). Mohammad et al. revealed that with no characteristic clinical symptoms of CN, a correct diagnosis can be made by radiographic imaging, histopathology assessment, and immunohistochemistry (8). Chang et al. analyzed 781 patients with cancer and revealed a 5-year OS rate of 87.2% (9). Gabriele et al. revealed that CN were consistent with a low-grade neuronal neoplasm of the central nervous system, especially extraventricular neurocytoma (EVN) (10).

To the best of our knowledge, only few studies on CN have been reported to date. Considering the rarity of this disease, we conducted a retrospective analysis of a relatively large sample size of patients with CN using the SEER database, which covered 30% of the US population. This study aimed to identify clinical prognostic factors affecting the OS in patients with CN and to determine independent prognostic factors in the subgroups of different treatment modalities.

### 4.1 Age, sex, and race

Approximately 25% of CN develops in adults in their 30s (5). The most common age of onset of CN and EVN is 20–34 years (11). In our study, patients ranged in age ranged from 0 to 85 years. Further, in the overall data, >50% of patients diagnosed with CN were aged 20–39 years.

Mattar et al. revealed that age was not a significant prognostic factor in 22 patients diagnosed with atypical CN between January 2009 and March 2018. After reviewing the literature, the previous study concluded that neither age nor sex had a significant effect on the median OS (12–15).

In our univariate and multivariate analyses of 413 patients, age was not a significant prognostic factor. Further, all subgroup analyses revealed that age was not a significant factor affecting prognosis, which is consistent with the results of previous reports. In the subgroup analysis of patients with STR and those who received RT, men showed better outcomes than women. The subgroup analysis of patients with STR revealed that African American had a better prognosis than other races.

### 4.2 Tumor size

In a retrospective analysis of 868 neurocytomas, Dutta et al. revealed that the median tumor size was 4–5 cm and that tumor size was not a determining factor. Even patients with a tumor size of >4 cm had a 5-year OS rate of 89%. Furthermore, patients with GTR had a 5-year OS rate of 96% (16).

Our study revealed that HR increased with tumor size. In the no surgery and no RT subgroups, patients with a tumor size of >4 cm had a higher HR than those with a tumor size of <2 cm, indicating that tumors with a size of >4 cm had a lower survival rate than smaller tumors. This is also consistent with the general tumor growth pattern. Larger tumors are more likely to invade surrounding brain tissues, nerves, and the vascular system. Further, larger tumors are more difficult to treat surgically and are more likely to have residual tumor tissues and recurrence after surgery.

### 4.3 Primary site (tumor location)

EVNs can occur in any brain tissue except the ventricle. They are broad-spectrum, more aggressive, and have a worse prognosis (5, 17, 18). Joonho et al. revealed that EVN may be a heterogenous disease entity and needed to be followed up for a long time (19). Shuran et al. revealed that an accurate diagnosis was difficult to be made preoperatively in 11 patients with EVNs. When the imaging findings are atypical, more aggressive treatment should be considered in patients (20). Treatment options and prognosis vary widely between CN and other ventricular tumors (21). According to our RT subgroup analysis, CN located outside the ventricle had a worse prognosis.

### 4.4 Primary site surgery (therapy)

Currently, surgery is considered the gold standard for treating CN. Han et al. conducted a single-center study involving 67 patients and found that complete tumor resection was the preferred treatment (22). In particular, patients with GTR have a favorable prognosis and a significantly lower risk of CN recurrence. In a study involving 310 patients with CN, the 5-year OS rate of patients with GTR was as high as 99% (5, 23, 24). Mattar et al. conducted a retrospective analysis of 22 cases and concluded that GTR was an independent prognostic factor for OS in patients with CN (12). Liang et al. revealed that surgery can benefit children and ensure relatively long-term progression-free survival in 14 patients with pediatric CN (25). Qiongxuan et al. revealed that use of GTR whenever possible and close imaging follow-up in 101 patients with CN (26).

Alqroom et al. used the transcortical and interhemispheric transcallosal approaches in 18 and 14 patients with CN, respectively, and found no difference in the scope of resection or protection of nerve function between the two surgical approaches (2, 27). Further, according to Sing et al., intraoperative neuroelectrophysiological monitoring is important for safe lesion resection (28).

According to a systematic review by Mahavadi et al., in cases of a high risk of GTR, maximal safe resection combined with adjunct RT can be used as a suboptimal treatment alternative for cancer (29).

However, in a retrospective analysis of 868 neurocytomas, Dutta et al. revealed that the extent of resection was not an independent prognostic factor for improved survival using multivariate analysis.

In our multivariate regression analysis, GTR (HR: 0.298, 95% CI: 0.122–0.728, *P* = 0.008; **Table 2**) was an independent prognostic factor for OS. We found that no surgery, biopsy, surgery, NOS, and STR subgroups were associated with a worse prognosis than the GTR subgroup. In the no RT subgroup, patients with GTR showed a better prognosis. The therapeutic effect of GTR on CN has been fully confirmed in previous studies. GTR should be performed while preserving as many important physiological structures as possible.

### 4.5 Radiotherapy

Adjunct RT, such as stereotactic radiosurgery (SRS) and fractionated RT, plays an important role in the treatment of CN (5, 30–32).

According to the findings of Han et al., RT is not recommended following complete tumor resection. After the complete excision of the atypical CNs, adjuvant RT was not recommended, and close radiographic follow-up was required (22). In patients with incomplete tumor resections, adjuvant RT should be advocated (27); moreover, postoperative RT can improve OS in these patients.

Nakamura et al. argued that SRS is an effective method for treating recurrent or residual CNs after STR. Meanwhile, Gamma knife surgery plays an essential role in the postoperative treatment of patients with CN (30). There are no specific SRS dosage guidelines for CN treatment. Lee et al. and Matsunaga et al. recommended that a minimum of 13 Gy is required for effective tumor control (5, 33). Bui et al. and Minniti et al. suggested that an RT dose between 13 and 18 Gy is relatively safe (31, 34). They examined 150 cases and found that RT had >90% local tumor control and that radiotoxicity was uncommon (31). In addition, RT is associated with delayed complications and radiation-induced toxicity, including leukoencephalopathy, radiation-induced malignancy, and radiation necrosis (14, 30, 32).

Dutta et al. conducted a retrospective analysis of 868 cases of CN and revealed that RT was not a vital prognostic factor using multivariate analyses (16, 35). Furthermore, Dutta et al. and Hussain et al. reported that adjuvant RT did not significantly improve the OS rate of patients with CN and that the effect of salvage RT was unknown (16, 36). Dan et al. revealed that postoperative RT also did not improve local control and survival in 43 patients with CN (37). By studying 68 patients with CN, Lei She et al. revealed that postoperative RT could improve Progression-free survival (PFS) in STR, but not in OS (38). Göktug et al. reported that use of RT as a primary or adjuvant treatment following surgical resection remained controversial in a study of 25 CNs (39).

In our study, the role of RT in treating patients was crucial. However, a multivariate analysis of all patient data revealed that RT may reduce the OS rate of patients. In a subgroup analysis, the RT did not significantly improve the prognosis of patients with GTR. RT was not recommended after complete tumor resection. In the RT subgroup, patients with tumors outside the ventricle or women have a poorer prognosis than those with tumors within the ventricle or men, respectively. This suggests that RT is recommended for men or those with tumors located within the ventricle.

This result may be attributed to the limitations of the SEER database and the insufficient sample size. Our findings suggested that the patient’s condition should be thoroughly assessed prior to RT. Physicians should consider RT toxicity and the harm caused by subsequent cognitive decline to patients (16). GTR or RT may impair important brain structures and functions, leading to a decline in quality of life. Extent of tumor resection and adjuvant treatments should always be balanced between prognosis improvement and maintenance/worsening of quality of life.

### 4.6 Chemotherapy

Currently, chemotherapy for treating patients with CN is controversial, with no corresponding treatment guidelines (40). According to Dutta et al., chemotherapy might be considered when patients are unable to complete surgery or RT. However, the most effective chemotherapy drugs are yet to be identified (16).

Johnson et al. conducted a retrospective analysis of 39 cases of CN treated with chemotherapy and concluded that there is significant heterogeneity in chemotherapy for CN. Furthermore, they emphasized that the benefits of temozolomide for treating CN are unclear and need further investigation (40). There are no prospective, multicenter, large-scale studies on chemotherapy for CN. In the multivariate regression analysis and the five treatments subgroup analysis, chemotherapy was not an independent prognostic factor for OS. Finally, only six patients completed chemotherapy, indicating that the efficacy of chemotherapy requires further investigation.

## 5 Limitations

Due to multiple changes in the diagnostic criteria for CN between 2000 and 2019, there was heterogeneity among patients included in the SEER database. In other words, there was a particular patient selection bias based on the SEER database. In our study, after data cleaning, there were no patients with malignant CN. The limitation of the article mentioned that our study lacked immunohistochemical data. In addition, the sample size is relatively small in this study. Longer follow-up and further multicenter studies with more sample sizes are needed.

## 6 Conclusion

In our study, patients with small tumors or GTR or those who did not receive RT showed a better prognosis. GTR was the preferred treatment for CN. RT was not recommended for patients after GTR. Men and African American showed certain advantages after STR surgery. Tumors with a size of >4 cm were recommended for active treatment. In the RT subgroup, patients with tumors outside the ventricle or women had a poorer prognosis than those with tumors within the ventricle or men, respectively. These findings will help clinicians and patients understand the treatment and prognosis of CN visually and intuitively.

## Data availability statement

The datasets presented in this study can be found in online repositories. The names of the repository/repositories and accession number(s) can be found in the article/supplementary material.

## Ethics statement

Ethical review and approval was not required for the study on human participants in accordance with the local legislation and institutional requirements. Written informed consent from the participants’ legal guardian/next of kin was not required to participate in this study in accordance with the national legislation and the institutional requirements. Written informed consent was obtained from the individual(s), and minor(s)’ legal guardian/next of kin, for the publication of any potentially identifiable images or data included in this article.

## Author contributions

RZ and ZZ: conceived the design of the study. XJ and JY: perfected the researched idea and obtained detailed data resources. CZ, XP and YW: used and implemented statistical methods to data. QL, HC and ZZ: finished the preliminary writing of the paper. †: these authors contributed equally to this work. *: corresponding author. All authors: offered valuable advice and confirmed the final revision of the article. All authors contributed to the article and approved the submitted version.
